# Treatment of Prolapsus of the Funus

**Published:** 1860-05

**Authors:** 


*Treatment of Prolapsus of the Funus.*—Prolapsus ot the funus is not an uncommon accident, and, without appropriate treatment, it is one that often results unfortunately to the life of the child and the hopes of the mother. Professor Mendenhall, of Cincinnati, applauds the treatment of this accident by the position of the mother; successful cases have been reported. In the "Lancet and Observer," for January, he reports another case, converted in a few moments, by this method, into a case of simple labor. He places the woman on her breast and knees, in which position the funis is readily replaced. The position may be maintained, if need be, until the presenting part occupies the pelvic strait. It is probable that the position need not be long maintained. Prof. Mendenhall concludes his paper with the following remark: "In view of the frequent fatality to the child of this complication, I deem a knowledge of its proper treatment a matter of great importance. I think with this knowledge that few, if any cases ought to result unfavorably to the child, and a resort to turning the child is seldom, if ever, necessary."

---

*Arsenical Poisoning by Paper Hangings.*—Three children near Tipton have suffered from the arsenical emanations from a green bed room paper in a newly papered house. The symptoms were emaciation, pining, general restlessness, and twitching of the facial muscles. Dr. Belenden, observing these symptoms, concluded that they were suffering from the gradual effects of poisoning: and on being removed into another room, the children recovered. *London Lancet.*

